# Containing Carbapenemase-producing *Klebsiella pneumoniae* in an endemic setting

**DOI:** 10.1186/s13756-020-00766-x

**Published:** 2020-07-06

**Authors:** Kalliopi Spyridopoulou, Mina Psichogiou, Vana Sypsa, Vivi Miriagou, Amalia Karapanou, Linos Hadjihannas, Leonidas Tzouvelekis, George L. Daikos

**Affiliations:** 1 First Department of Medicine, Medical School, “Laiko” General Hospital, National and Kapodistrian University of Athens, 75, 11527 Athens, Greece; 2 Department of Hygiene, Epidemiology and Medical Statistics, Medical School, National and Kapodistrian University of Athens, Athens, Greece; 3 Laboratory of Bacteriology, Hellenic Pasteur Institute, Athens, Greece; 4 Department of Microbiology, School of Medicine, National and Kapodistrian University of Athens, Athens, Greece

**Keywords:** Carbapenemases, Infection control, *Klebsiella pneumoniae*, Hospital-acquired infection, Active surveillance

## Abstract

**Background:**

Carbapenemase-producing *K. pneumoniae* (CP-Kp) has been established as important nosocomial pathogen in most tertiary care hospitals in Greece. The aim of the present study was to examine the impact of an enhanced infection control program on the containment of CP-Kp in a haematology unit where the incidence of CP-Kp infections was high.

**Methods:**

The study was conducted from June 2011 to December 2014 in a haematology unit of a tertiary-care 500-bed hospital located in Athens, Greece. A bundled intervention (active surveillance cultures, separation of carriers from non-carriers, assignment of dedicated nursing staff, contact precautions, environmental cleaning, and promotion of hand hygiene) was tested whether would reduce colonization and infection caused by CP-Kp.

**Results:**

A total of 2507 rectal swabs were obtained; 1199 upon admission from June 2011 to June 2013 and 1307 during hospitalization from June 2011 to December 2012. During intervention the admission prevalence of CP-Kp colonization (*p* < 0.001 for linear trend), the hospitalization prevalence (*p* = 0.001 for linear trend) and the incidence rate of CP-Kp colonization (*p* = 0.072 for linear trend) were declining. Application of segmented linear regression revealed that both the change in the level of CP-Kp BSI incidence rates (*p* = 0.001) as well as the difference between pre- and post-intervention slopes were statistically significant (*p* < 0.001).

**Conclusions:**

A bundled intervention including active surveillance cultures on admission can attain maximum containment of CP-Kp colonization and infection in endemic acute healthcare settings.

## Background

Carbapenemase-producing *K. pneumoniae* (CP-Kp) has been established as important nosocomial pathogen worldwide [1, 2]. A high prevalence of CP-Kp is now observed in many countries causing serious infections that are associated with prolonged hospitalization and increased morbidity and mortality [3, 4]. In healthcare facilities with inadequate infection control practices, these organisms spread from patient to patient very efficiently. The number of new cases generated by a primary case, as represented by the basic reproduction number *R*_*0*_, is estimated to be 2, a number sufficient to trigger outbreaks in the healthcare setting [5]. In addition, transfer of patients between institutions in the same region and/or across borders from high- to low-prevalence countries enables further dispersion of these organisms.

Infection and colonization rates by CP-Kp strains in Greek tertiary care hospitals are considered amongst the highest in Europe [3]. The dire consequences in affected patients plus the widely held aspect that transfer of infected/colonized patients from Greece is an important factor for the spread of CP-Kps in Northern Europe [4, 6], have led to repeated attempts to curb the problem. Yet, the relevant surveillance records clearly show that the success of the hitherto policies has been marginal and short-lived [7].

Recognizing the urgent need to reduce the dispersion of CP-pathogens, the Hellenic Center for Disease Control and Prevention has drawn up an action plan for the containment of infections caused by multi-drug resistant organisms in health-care facilities [8]. The basic aim of this plan is to implement enhanced infection control measures in all healthcare facilities in order to contain the spread of carbapenem resistant *Klebsiella spp*, *Acinetobacter spp* and *Pseudomonas spp.* However, the escalating shortages in human and financial resources in many of our hospitals do not allow the full and proper implementation of the required measures in a sustainable and timely fashion [9].

The application of mathematical modeling in the data of a previous observational study conducted in a unit of our hospital (Laiko General Hospital, Athens, Greece) where CP-Kp endemicity was sustained by influx of carriers and cross-transmission, indicated the pivotal role of active surveillance cultures in controlling CP-Kps [5]. Based on the above findings and taking into account the existing shortages, we attempted to contain CP-Kp spread by applying a bundle of feasible measures, including active screening, in the haematology unit where the incidence of CP-Kp infections and the associated mortality were high. Herein, we present the design and the results of this intervention.

## Methods

### Setting

The study was conducted from June 2011 to December 2014 in a 31-bed haematology unit of a tertiary-care 500-bed hospital located in Athens, Greece. The unit is consisted of fourteen rooms; seven 3-bed, three 2-bed and four single rooms. The vast majority of patients admitted in the unit are patients with haematologic malignancies. The mean nurse to patient ratio was 1:10 during the morning shift and 1:15 during afternoon and night shifts. Compliance of healthcare workers (HCWs) with hand hygiene before intervention was ranging from 10 to 20%. After January 2011 an increasing number of CP-Kp BSIs was observed in the unit. A point prevalence for rectal colonization was conducted among 28 patients hospitalized in the unit which revealed that 8 (28.6%) patients were colonized with CP-Kp. These observations prompted the infection control committee to design and implement an enhanced infection control program.

### Intervention

**Fig. 1:**
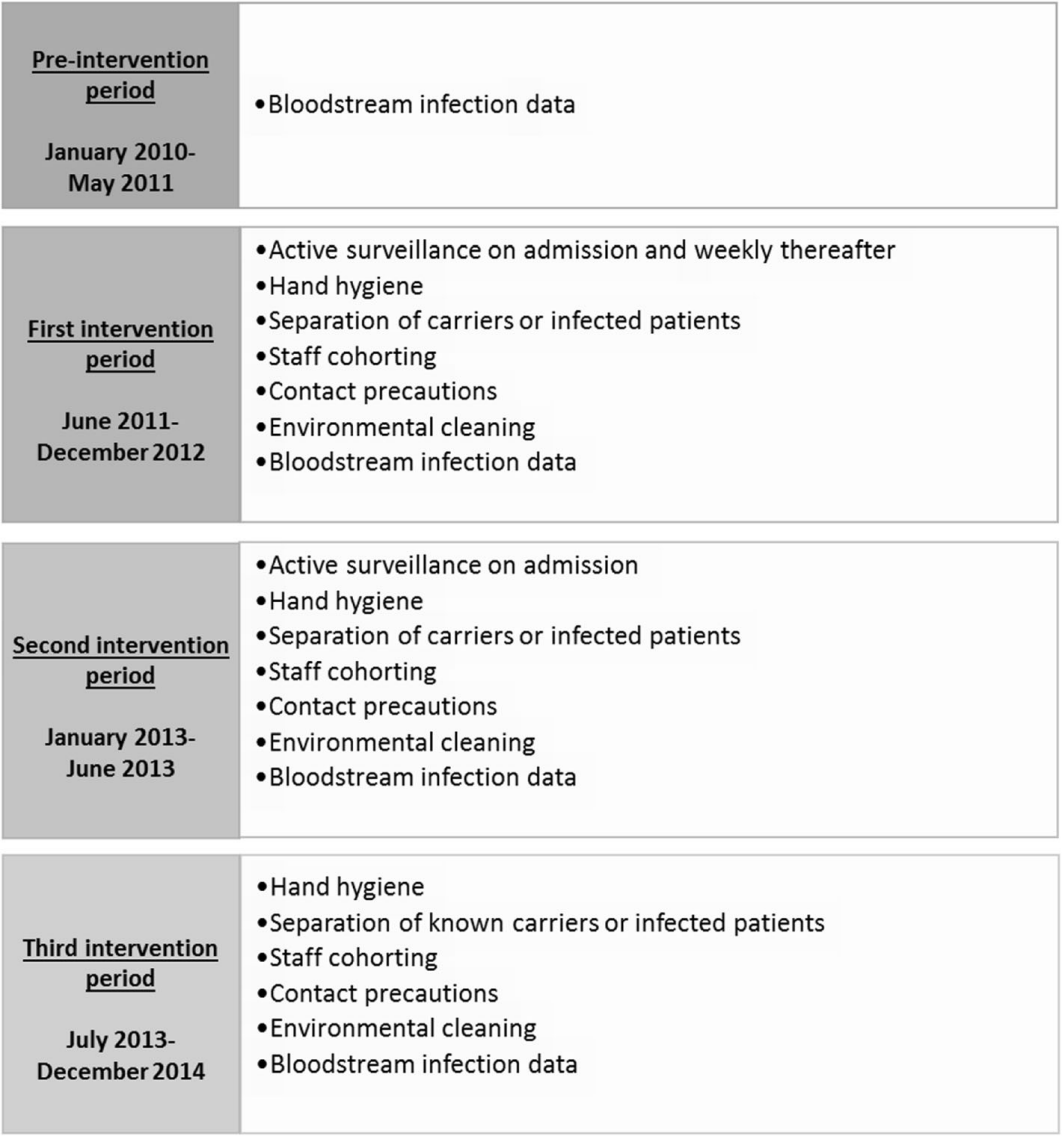
Intervention measures according to time period

Before implementing the bundled intervention in June 2011, all healthcare personnel attended an educational course. Training and feedback were provided from the infection control team once a month initially and every 3 months thereafter. Hand-washing basins and dispensers containing alcohol-based disinfectants were located near all beds. Personal protective items including gloves and gowns and dedicated patient-care equipment were made available in the unit. Also, written instructions were given to all patient relatives. The infection control measures included i) hand hygiene, ii) active surveillance cultures of rectal swabs within 24 h after admission and weekly thereafter for all patients staying for more than 24 h, iii) separation of carriers or infected patients in single rooms or cohorting in two- or three-bed rooms, iv) staff cohorting to care for patients known to harbour CP-Kp, when feasible, v) contact precautions, and vi) environmental cleaning of patient-room twice daily. The weekly follow-up surveillance cultures during hospitalization were performed until the CP-Kp transmission was considered negligible (December 2012) and the surveillance cultures on admission were continued up to June 2013 (Fig. 1). All patients admitted to the unit during the study period were recorded in a database along with admission and discharge dates and information concerning colonization and /or infection during hospitalization. The data from the active surveillance were recorded in a separate database along with data on the phenotypic and molecular characteristics of CP-Kp isolates. In addition, data on bloodstream infections (BSIs) were collected retrospectively from January 2010 to May 2011 and prospectively from June 2011 to December 2014.

### Monitoring for compliance

Hand hygiene compliance was estimated by direct unobtrusive observation of patient-HCW contacts. Observations of 20–30 min each were performed by a trained observer who recorded health care activities. More specifically, during the observation-sessions, the observer recorded the opportunities for hand hygiene and the action performed by the HCW as either action performed (rubbing with an alcohol-based hand rub, washing with soap and water, both washing and rubbing) or not performed, according to the “My five moments for hand hygiene” WHO Hand Hygiene Improvement Strategy. A mean of 200 observations were performed every month. Compliance to isolation (single room or cohorting) of colonized/infected patients, the assignment of dedicated nursing staff for carriers, as well as the implementation of contact precautions were monitored daily.

### Microbiology

Rectal swabs were obtained and inoculated on McConkey agar No.3 plates (OXOID) containing 0.5 mg/L of meropenem and incubated at 37 °C for a maximum of 48 h. Isolates identified as *K. pneumoniae* were examined for production of carbapenemases by combined disk synergy test [10]. Carbapenemase encoding genes (*bla*_VIM_, *bla*_KPC_, *bla*_NDM,_ and *bla*_OXA-48_) were detected by polymerase chain reaction and the genetic relatedness of the isolates was examined by pulsed-field gel electrophoresis (PFGE), as described previously [11].

### Definitions and study outcomes

As primary outcome of the intervention was the incidence of CP-Kp BSIs. Secondary outcomes included the incidence and prevalence of CP-Kp colonization of hospitalized patients, the admission prevalence of CP-Kp colonization and the incidence of BSIs due to any pathogen. A patient was defined as colonized if he/she was found positive for CP-Kp in a surveillance culture. A patient found positive in the first surveillance culture upon admission was considered to be already colonized before entering the unit, whereas when a patient found CP-Kp negative upon admission and positive in any of the follow-up rectal swab cultures was considered as being colonized during hospitalization. A colonized patient was thought to be decolonized if the investigators had recorded at least three consecutive rectal samples to be CP-Kp negative. CP-Kp BSI was defined as detection of CP-Kp in a blood culture along with consistent clinical findings. Onset of BSI was defined as the date of collection of the first blood culture that yielded CP-Kp.

### Statistical analysis

CP-Kp colonization on admission was assed based on rectal swabs obtained within 24 h since patient’s admission. The admission prevalence of CP-Kp colonization per month was then estimated as the proportion of positive rectal swabs out of the total number of swabs collected upon admission during that month. We also estimated the hospitalization prevalence of CP-Kp colonisation per month as the proportion of colonised patients out of the total number of hospitalised patients in the unit during that month (patients with at least one positive swab were considered colonized). We assessed the post-intervention slope of the linear trend in the monthly CP-Kp colonisation prevalence on admission and during hospitalisation using linear regression. We estimated the incidence rate of CP-Kp colonization during hospitalization per month. Only patients who were not colonized on admission and had at least two available swabs per admission, one upon admission and at least one during hospitalization, were included in the analysis. The person-time was calculated as the time between admission and discharge for those who remained un-colonised, or as the time between admission and the midpoint between the last negative and first positive swab for those who were colonized during hospitalization. The incidence rate of colonization was then estimated as the number of new colonisations occurring during hospitalization divided by the total person-time at risk for that one-month period. We further estimated the incidence of BSIs (bimonthly or quarterly) as the number of new infections occurring in the specific period over the total patient-time at risk in that period. Similarly, we assessed the incidence of bloodstream infections caused by pathogens other than CP-Kp. As in the case of CP-Kp BSI incidence, there were data available during a long pre-intervention period. Thus, it was possible to assess the impact of the intervention using the longitudinal data series on BSI incidence before and after its implementation in June 2011 using segmented linear regression. This method allowed to estimate the change in level of BSI incidence rates immediately after the intervention and the difference between pre- and post-intervention slopes of the outcome trend.

## Results

### Compliance to intervention measures

A total of 2005 patients were admitted in the unit; 1199 from June 2011 to June 2013 and 806 from July 2013 to December 2014. The mean hospital stay was 15 days and the median 9 days. Overall, 2507 rectal swabs were obtained; 1199 upon admission from June 2011 to June 2013 and 1307 during hospitalization from June 2011 to December 2012. The average post-intervention monthly compliance with hand hygiene among the medical and nursing staff was 60 and 50%, respectively (number of performed actions, 4029). High level of adherence was observed in isolation/cohorting (100% of CP-Kp colonized/infected patients) and in contact precautions (100%). Given the low nurse to patient ratio, dedicated nursing staff was assigned only during the morning shift.

### Impact of intervention on CP-Kp colonization and infection

**Fig. 2:**
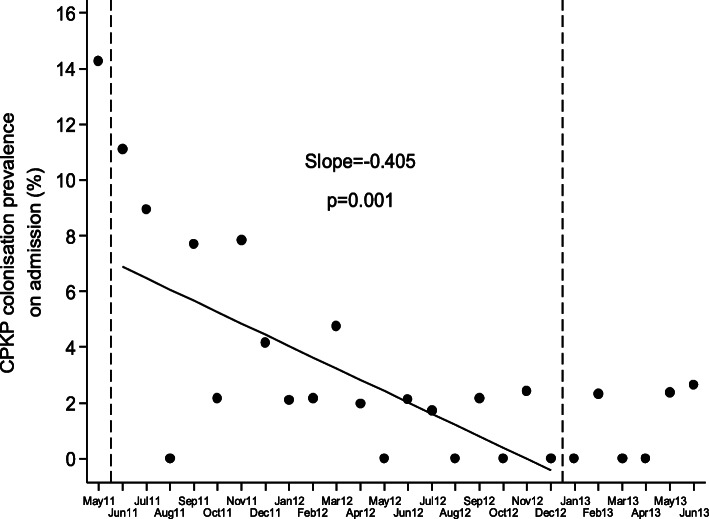
Monthly prevalence of CP-Kp colonization on admission during three periods indicated by the vertical dashed lines: May 2011 (pre-intervention), June 2011–December 2012 (implementation of the intervention including surveillance cultures obtained on admission, weekly and on discharge) and January–June 2013 (surveillance cultures only on admission). The solid circles dots indicate the observed monthly prevalence on admission and the dashed line is the corresponding fitted regression line for the period June 2011–December 2012

**Fig. 3:**
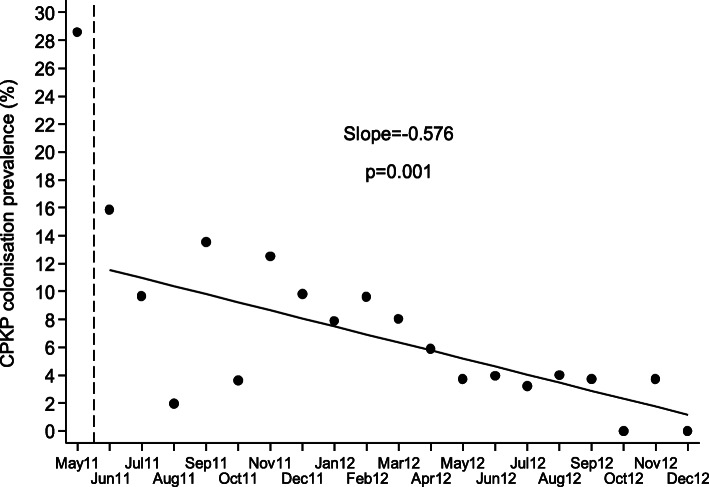
Monthly prevalence of CP-Kp colonization. The vertical dashed line indicates the initiation of the intervention (June 2011). The solid circles indicate the observed monthly prevalence and the solid line is the corresponding fitted regression line for the period June 2011–December 2012. After December 2012, only prevalence on admission was assessed

**Fig. 4:**
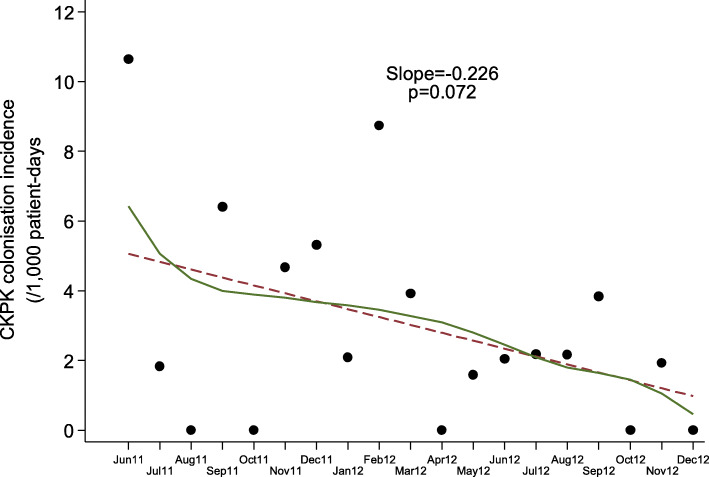
Incidence rate of CP-Kp colonization per month (solid circles: monthly incidence rates, solid line: lowess smoothing, dashed line: fitted linear regression line)

**Fig. 5:**
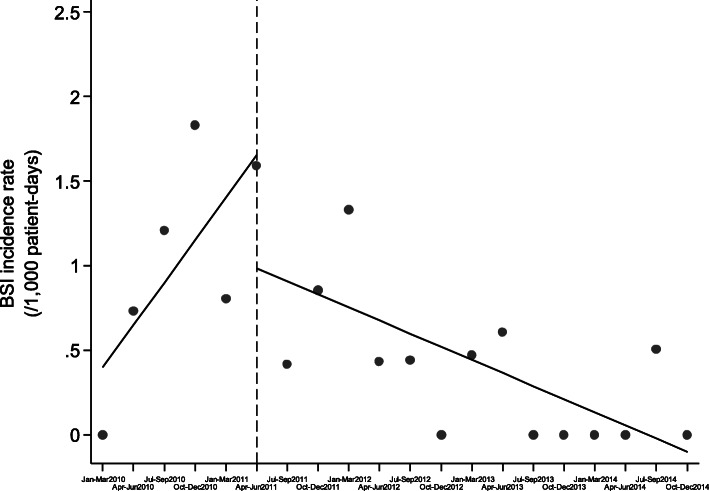
BSI incidence rate during 2010–2014 (solid circles: BSI incidence rates per 3-month periods, dashed vertical line: initiation of intervention, solid lines: fitted regression lines for the periods pre- and post-intervention)

**Fig. 6:**
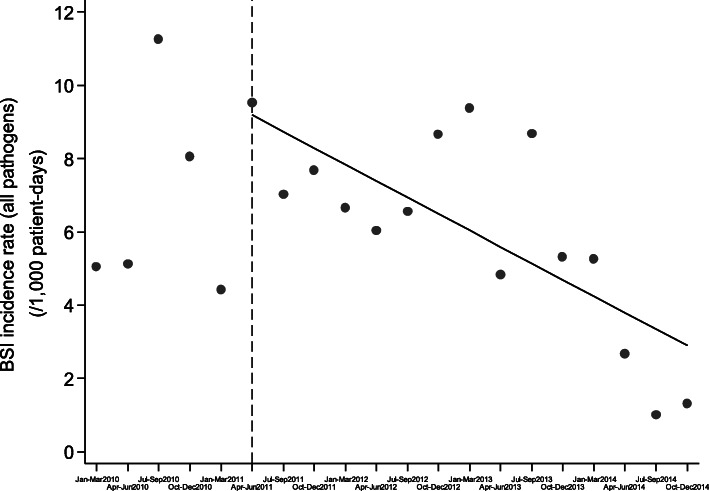
or supplementary. BSI incidence rate (all pathogens) during 2010–2014 (solid circles: BSI incidence rates per 3-month periods, dashed vertical line: initiation of intervention, solid lines: fitted regression line)

Although continuous inflow of already colonized patients was observed during the post-intervention period, the monthly prevalence of CP-Kp colonization on admission was declining (slope of linear trend [95% CI]: − 0.405 [− 0.627, − 0.182], *p* < 0.001) and the number of new CP-Kp carriers decreasing (Fig. 2). Also, as shown in Fig. 3, the monthly prevalence of CP-Kp colonization among hospitalized patients progressively decreased from 15.9% in June 2011 to 0% in December 2012 (slope of linear trend [95% CI]: − 0.576 [− 0.863, − 0.289], *P* value = 0.001). The colonization incidence during hospitalization was 10.6/1000 patient-days in June 2011 and declined to 0/1000 patient-days in December 2012 (slope of linear trend [95% CI]: − 0.227 [− 0.476, 0.023], *p* = 0.072), indicating negligible cross transmission (Fig. 4). More importantly, as shown in Fig. 5, the intervention measures halted the increasing incidence of CP-Kp BSIs observed in the pre-intervention period and eventually resulted in decline from 1.58/1000 patient-days in April–June 2011 to 0/1000 patient-days in July–September 2013 and remained so until December 2014, with the exception of one patient with CP-Kp BSI transferred from another hospital. The application of segmented linear regression in bimonthly BSI incidence rates revealed that both the change in the level of BSI incidence rates (i.e. the impact of the intervention) as well as the difference between pre- and post-intervention slopes were statistically significant (coefficients [95% CI]: − 1.545 [− 2.388, − 0.702] *p* = 0.001 and − 0.332 [− 0.502, − 0.162], *p* < 0.001, respectively). Moreover, a decline in the incidence of BSIs caused by pathogens other than CP-Kp was observed (slope of linear trend in quarterly incidence post-intervention [95% CI}: − 0.150 (− 0.231, − 0.06 [9]], *p* = 0.012) (Fig. 6). Of note, 11 (28.9%) of 38 (8 found in point prevalence survey, 13 on admission and 17 during hospitalization) colonized patients developed BSI caused by CP-Kp, of whom seven (63.6%) died due to sepsis, while none of the non-carriers developed any kind of CP-Kp infection.

### Microorganisms

Thirty-one of 40 colonizing organisms (two patients were colonized with two different strains during different hospitalization periods) produced *Klebsiella pneumoniae* carbapenemase (KPC), 7 Verona integron-encoded metallo-beta-lactamase (VIM), one New Delhi metallo-beta-lactamase (NDM-1) and one isolate both type of enzymes, VIM and KPC. Regarding the BSI isolates, nine produced KPC, one VIM and one NDM. A total of 67 *K. pneumoniae* isolates were studied by PFGE, 57 obtained from surveillance cultures (more than one isolates per patient were available from 18 of 38 carriers) and 10 from blood cultures. Fifty-four organisms produced KPC, nine VIM, three NDM and one isolate, both type of enzymes, VIM and KPC. PFGE typing revealed 15 different genomic profiles. Forty-six of 54 KPC isolates were grouped in one PFGE type, while the remaining 8 isolates displayed six distinct profiles containing 1 to 2 isolates each. The nine VIM-producers exhibited seven different PFGE types, each one containing 1 to 2 isolates and the three NDM-producers consisted a distinct group. The isolate that carried both *bla*_VIM_ and *bla*_KPC_ genes was classified in one of the KPC PFGE types. Of note, all BSIs were caused by CP-Kp exhibiting identical PFGE type with that of the colonizing organism (Table 1).

**Table 1:** Characteristics of *Klebsiella pneumoniae* isolates.

**Microorganisms**	**Carbapenemase type**				
	**Site of isolation**	**KPC**	**VIM**	**NDM**	**KPC+VIM**
	**Rectum**	A (*n*=25)	H (*n*=1)		
		B (*n*=1)	I (*n*=1)		
		C (*n*=1)	J (*n*=1)		
		31 D (*n*=1)	7 K (*n*=1)	1 O (*n*=1)	1 E (*n*=1)
		E (*n*=1)	L (*n*=1)		
		F (*n*=1)	M (*n*=1)		
		G (*n*=1)	N (*n*=1)		
	**Blood**	8 A (*n*=8)	1 M (*n*=1)	1 O (*n*=1)	-

## Discussion

The findings presented herein demonstrate that early identification of asymptomatic CP-Kp carriers followed by physical separation from non-carriers in conjunction with contact precautions, improved compliance in hand hygiene, and environmental cleaning, had a significant effect on containing CP-Kp spread and on reducing CP-Kp BSIs in an haematology unit where the incidence of CP-Kp infections and the associated mortality were high.

Prior to intervention a substantial proportion (28.6%) of hospitalized patients in the unit were colonized with CP-Kp. Apparently, this was result of i) constant inflow of CP-Kp colonized patients and ii) transmission from patient to patient due to inadequate infection control practices. Indeed, approximately 10% of the admitted patients were already colonized and a large proportion of CP-Kp isolates examined by PFGE was classified in groups with identical genetic profiles, indicating ongoing horizontal transmission. In health care facilities with such characteristics, the infection control strategy should aim to interrupt both chains of transmission: i) the inflow of carriers by early identification and physical separation and ii) the horizontal transmission by contact precautions and enhanced standard infection control practices.

Transmission from patient to patient via the hands of health care workers is the main route of spread in the acute-care setting [12]. In a modeling study, performed in our hospital, the minimum hand hygiene compliance level required to control CP-Kp transmission was estimated to be 50% [5]. This target was achieved in our unit, however, in settings with low nurse to patient ratio, it is questionable whether a substantial improvement in hand hygiene can be achieved, and if so, whether such an improvement could be sustained over time. Moreover, the hand hygiene alone, even at high compliance rates, had poor impact on the spread of CP-Kp in our hospital where there was constant inflow of new colonized patients [5]. Thus, in endemic settings additional measures are required to contain CP-Kp as has been recommended by different organizations, scientific societies and experts [13–15].

Unrecognized CP-Kp carriers can serve as a potential source of transmission to other patients. Previous studies have shown that the use of clinical cultures may fail to detect a significant proportion of colonized patients and only after employing active surveillance the incidence of CP-Kp infections declined [16, 17]. Similarly, in the present study, active screening was considered to be of pivotal importance for several reasons. First, the recorded monthly prevalence of CP-Kp colonization on admission before the intervention was > 10% and ranged between 0 and 11.1% during the study period; thus, a significant number of CP-Kp-positive patients would had been unrecognized without active screening. Second, when a CP-Kp carrier was detected, the patient was isolated or cohorted and contact precautions were employed immediately. Finally, active screening provided information not only for the colonization status of each patient but it also served as an indicator to monitor the success of the implemented measures. In settings with no adequate resources, as in our unit, active screening can be discontinued when no further transmission occurs and screening on admission can be limited only to high-risk patients as defined by local epidemiology.

Containment of carbapenemase-producing Enterobactericeae (CRE) has been demonstrated by several studies after applying infection control interventions in the form of bundles [16, 18–25]. The design of these studies does not allow to evaluate the effectiveness of each intervention in controlling transmission. Several investigators, however, have implemented bundles in a stepwise approach and have indicated that early identification of asymptomatic carriers followed by separation of carriers and staff cohorting were very essential in the containment of CRE [26–29]. In line with these findings, soon after employing the infection control measures in our unit, the incidence and prevalence of colonization progressively declined. More importantly, in parallel with the decline of the newly colonized patients, a reduction in the incidence of CP-Kp BSIs and in the incidence of BSIs caused by other pathogens was observed.

Previous studies have shown that a substantial proportion of CP-Kp colonized haematologic patients may develop bacteremia with detrimental effects for the host [30–32]. In our unit, 11 (28,9%) of 38 colonized patients developed CP-Kp BSIs and 7 (63.6%) of those died due to infection. Of note, the strains causing bacteremia had identical PFGE pattern with the colonizers. Apparently, after ingestion and establishment of the organisms in the gastrointestinal tract, CP-Kp may migrate to clinical sites and cause infection with harmful effects for the host. Thus, it is of paramount importance to act pro-actively and protect these patients before they get colonized.

## Conclusion

Herein was shown that bundled intervention, including active surveillance, can attain maximum containment of CP-Kp colonization and infection in endemic acute healthcare settings. Considering the impact of CP-Kp infections on human lives, a multifaceted intervention programme to prevent and control such infections should be in place in all acute health care facilities. When a hospital-wide intervention is not feasible due to limited resources, there is need for prioritization and prompt implementation of a rigorous and effective program in “high risk” units to protect the most vulnerable patients.

## Data Availability

The datasets used and/or analyzed during the current study are available from the corresponding author on reasonable request.

## Abbreviations

CP-Kp: Carbapenemase-producing *Klebsiella pneumoniae*
BSIs: Bloodstream infections
HCWs: Health-care workers
WHO: World Health Organization
*bla*_VIM_: Verona Integron-Mediated Metallo-β-lactamase gene
*bla*_KPC_: *Klebsiella pneumoniae* carbapenemase gene
*bla*_NDM_: New Delhi Metallo-beta-lactamase gene
*bla*_OXA-48_: Oxacillinase-48 gene
PFGE: Pulsed-field gel electrophoresis
CI: Confidence Interval
CRE: Carbapenem-resistant Enterobacteriaceae
